# Frequent tRNA gene translocation towards the boundaries with control regions contributes to the highly dynamic mitochondrial genome organization of the parasitic lice of mammals

**DOI:** 10.1186/s12864-021-07859-w

**Published:** 2021-08-06

**Authors:** Wen-Ge Dong, Yalun Dong, Xian-Guo Guo, Renfu Shao

**Affiliations:** 1 Institute of Pathogens and Vectors, Key Laboratory for Preventing and Controlling Plague in Yunnan Province, Dali University, 671000 Dali, China; 2 GeneCology Research Centre, University of the Sunshine Coast, Maroochydore, Queensland Australia; 3 School of Science, Technology and Engineering, University of the Sunshine Coast, Maroochydore, Queensland Australia

**Keywords:** tRNA translocation, Genome fragmentation, Mitochondrial karyotype, Parasitic lice

## Abstract

**Background:**

The typical single-chromosome mitochondrial (mt) genome of animals has fragmented into multiple minichromosomes in the lineage Mitodivisia, which contains most of the parasitic lice of eutherian mammals. These parasitic lice differ from each other even among congeneric species in mt karyotype, i.e. the number of minichromosomes, and the gene content and gene order in each minichromosome, which is in stark contrast to the extremely conserved single-chromosome mt genomes across most animal lineages. How fragmented mt genomes evolved is still poorly understood. We use *Polyplax* sucking lice as a model to investigate how tRNA gene translocation shapes the dynamic mt karyotypes.

**Results:**

We sequenced the full mt genome of the Asian grey shrew louse, *Polyplax reclinata*. We then inferred the ancestral mt karyotype for *Polyplax* lice and compared it with the mt karyotypes of the three *Polyplax* species sequenced to date. We found that tRNA genes were entirely responsible for mt karyotype variation among these three species of *Polyplax* lice. Furthermore, tRNA gene translocation observed in *Polyplax* lice was only between different types of minichromosomes and towards the boundaries with the control region. A similar pattern of tRNA gene translocation can also been seen in other sucking lice with fragmented mt genomes.

**Conclusions:**

We conclude that inter-minichromosomal tRNA gene translocation orientated towards the boundaries with the control region is a major contributing factor to the highly dynamic mitochondrial genome organization in the parasitic lice of mammals.

**Supplementary Information:**

The online version contains supplementary material available at 10.1186/s12864-021-07859-w.

## Background

Extensive fragmentation of mitochondrial (mt) genome was discovered first in three species of human lice, in which the single mt chromosome typical of animals evolved into 14 and 20 minichromosomes; each minichromosome has 1–5 genes and is 1.8–4 kb in size [1, 2]. Since then, 26 more species of parasitic lice have been sequenced for mt genomes [3–12]. It appears that mt genome fragmentation occurred at least twice in parasitic lice: once 60–90 million years ago (MYA) in the most recent common ancestor of Mitodivisia - a newly identified clade that contains the vast majority of parasitic lice of eutherian mammals [10, 13–15], and later ~ 25 MYA in the feather lice of the genus *Columbicola* [11, 13].

Unlike the extremely conserved single-chromosome mt genomes in most animal lineages [16] but somehow resembling the nuclear genomes of eukaryotes [17–19], the mt genome organization is highly dynamic in parasitic lice of the Mitodivisia clade [10]. These lice differ from each other, even among closely related species in the same genus, in mt karyotype, i.e. the number of minichromosomes, and the gene content and gene order in each minichromosome [8, 12]. The pig lice, *Haemapinus apri* and *Haemapinus suis*, and the horse louse, *Haemapinus asini*, have nine minichromosomes [3, 6], whereas the human head louse, *Pediculus humanus capitis*, and the human body louse, *Pediculus humanus humanus*, have 20 minichromosomes [1, 2]. Between these extremes, the elephant louse, *Haematomyzus elephantis*, has 10 minichromosomes (four genes not identified) [8]. The rodent lice, *Polyplax asiatica*, *Polyplax spinulosa*, *Hoplopleura akanezumi* (nine genes not identified) and *Hoplopleura kitti* (three genes not identified), have 11 minichromosomes [4, 5]. The guanaco louse, *Microthoracius praelongiceps*, the sheep louse, *Bovicola ovis*, the cattle louse, *Bovicola bovis*, and the macaque louse, *Pedicinus obtusus*, have 12 minichromosomes [9, 10, 12]. The goat louse, *Bovicola caprae*, and the dog louse, *Trichodectes canis*, have 13 minichromosomes [10]. The human pubic louse, *Pthirus pubis* (one gene not identified), and the colobus louse, *Pedicinus badii*, have 14 minichromosomes [2, 12]. Each minichromosome has 1 to 8 genes; the arrangement of genes in a minichromosome varies from species to species even within a genus; the only exceptions are: (1) the human head louse and the human body louse in the genus *Pediculus*, which diverged 83,000-170,000 years ago at the origin of clothing [1, 2, 20–22]; and (2) the domestic pig louse and the wild pig louse in the genus *Haemapinus*, which diverged ~ 10,000 years ago when pigs were domesticated [3, 23].

Why is the fragmented mt genome organization so variable in these parasitic lice? A few comparative studies to date have suggested four models or mechanisms. First, Shao et al., (2012) [2] showed that point mutation at the third anti-codon position or homologous recombination between tRNA genes accounted for the swap of identity and location between *trnL*_*1*_ and *trnL*_*2*_ and between *trnR* and *trnG* in the human lice. Dong et al., (2014a) [4] confirmed that this mechanism accounted for the swap of identity and location between *trnL*_*1*_ and *trnL*_*2*_ in the spiny rat louse, *Polyplax spinulosa*. Second, Shao et al., (2012) [2] showed that one minichromosome could split into two via gene degeneration followed by deletion, based on analysis of pseudo gene sequences of the human pubic louse. Third, Song et al., (2014) [6] showed that inter-minichromosomal recombination accounted for gene translocation in the horse louse, *Haematopinus asini*, and mt karyotype variation among *Haematopinus* lice. Fourth, Shao et al., (2017) [9] showed that both split and merger of minichromosomes had occurred and contributed to the variation in mt karyotypes in sucking lice.

In the present study, we used *Polyplax* sucking lice, which parasitize rodents and shrews, as a model to investigate the role of tRNA gene translocation in shaping the dynamic mt karyotypes. We show that frequent directional tRNA gene translocation between different types of minichromosomes is a major contributor to the highly dynamic mt genome organization of the parasitic lice of mammals.

## Materials and methods

### Louse collection, DNA extraction, mitochondrial genome amplification and sequencing

Specimens of *Polyplax reclinata* (family Polyplacidae) were collected in Yunnan Province, China, from the Asian grey shrew, *Crocidura attenuata* (family Soricidae). *Polyplax reclinata* was identified according to Chin (1999) [24]; voucher specimens (# 364) were kept at the Institute of Pathogens and Vectors, Dali University (Additional file 1). Sucking lice collected on the body surface of *Crocidura attenuata* were preserved in 95 % ethanol at − 20 °C prior to DNA extraction. Capture of shrews was approved by health authorities in Yunnan Province, China. Animal capture protocols and procedures were approved by the animal ethics committees at Dali University. Genomic DNA was extracted from individual lice with DNeasy Blood & Tissue kit (QIAGEN). Two pairs of primers, 12SA–12SB [25] and 16SF–Lx16SR [9], were used to amplify fragments of mt genes *rrnS* (375 bp) and *rrnL* (360 bp) (Additional file 2). These primers target conserved gene sequence motifs among arthropods. The two gene fragments were sequenced directly using Sanger method at the Thermo Fisher Scientific Genome Sequencing Facility (Guangzhou). Two pairs of specific primers for *P. reclinata*, 12S364F-12S364R and 16S364F-16S364R, were designed from *rrnS* and *rrnL* sequences obtained (Additional file 2). The two specific primers in each pair go outwards with 15 and 21 bp in between, respectively. PCRs with these specific primers amplified the near full-length *rrnS* and *rrnL* minichromosomes of *P. reclinata* (Additional file 3A). The amplicons from *rrnS* and *rrnL* minichromosomes, 2.3 and 2.6 kb in size respectively, were sequenced using Sanger method to obtain the sequences of non-coding regions. Another pair of primers specific to *P. reclinata*, 364F–364R (Additional file 2), was designed from conserved sequences of the non-coding regions that flank the coding regions of the *rrnS* and *rrnL* minichromosomes. The PCR with the primer pair 364F–364R produced a mixture of amplicons ranging from 1.1 to 1.8 kb in size, expected from the coding regions of the entire set of mt minichromosomes of *P. reclinata* (Additional file 3A). These amplicons were sequenced from both ends (i.e. paired-end) with Illumina Miseq platform: insert size 400 bp, read length 250 bp. The PCR strategy used in this study was developed from our observations in previous studies that each mt minichromosome has a distinct coding region but also a well-conserved non-coding region [1–6, 9].

Takara Ex Taq was used in the initial short PCRs with the following cycling conditions: 94 °C for 1 min; 35 cycles of 98 °C for 10 s, 45 °C for 30 s, 72 °C for 1 min; and a final extension of 72 °C for 2 min. Takara LA *Taq* was used in the long PCRs with the cycling conditions: 94 °C for 1 min; 35 cycles of 98 °C for 10 s, 55–65 °C (depending on primers) for 40 s, 68 °C for 4 min; and a final extension of 72 °C for 8 min. Negative controls were executed with each PCR experiment. PCR amplicons were checked by agarose gel (1 %) electrophoresis; the sizes of amplicons were estimated by comparing with DNA markers. PCR amplicons were purified with Wizard SV Gel/PCR clean-up system (Promega). Sanger sequencing was done at the Thermo Fisher Scientific Genome Sequencing Facility (Guangzhou). Illumina sequencing was done at the Majorbio Genome Sequencing Facility (Shanghai).

### Assembly of sequence reads, gene identification and minichromosome verification

Illumina sequence reads (250 bp each) were assembled into contigs with Geneious 11.1.5 [26]. The assembly parameters were minimum overlap 150 bp and minimum identity 98 %. tRNA genes were identified using tRNAscan-SE [27] and ARWEN [28]. Protein-coding genes and rRNA genes were identified by BLAST searches of GenBank [29, 30]. Sequences were aligned with Clustal X [31]. The size and circular organization of each mt minichromosome of *P. reclinata* were verified by PCR using outbound specific primers designed from the coding region of each minichromosome (Additional file 2). The forward primer and reverse primer in each pair were next to each other with a small gap in between. PCRs with these primers amplified each circular minichromosome in full or near full length (Additional file 3B); these amplicons were also sequenced with Illumina Miseq platform to obtain the full-length sequence of the non-coding region of each minichromosome. PCR set-up, cycling conditions, agarose gel electrophoresis and size measurement were the same as described above. Negative controls were run for each PCR test. The annotated mt genome sequence of *P. reclinata* was deposited in GenBank (accession numbers MW291451-MW291461).

### Phylogenetic analyses

We retrieved the mt genome sequences of all of the 14 species of sucking lice available in GenBank and the elephant louse (Table 1) and combined these sequences with that of *P. reclinata* generated in the present study. The elephant louse was used as the outgroup because it was in the suborder Rhynchophthirina, which was most closely related to sucking lice, the suborder Anoplura [10]. The sequences of eight mt protein-coding genes (*atp6*, *atp8, cox1*, *cox2*, *cox3, cob*, *nad4L*, *nad6*) and two rRNA genes (*rrnS* and *rrnL*) of these lice were used in phylogenetic analysis. Other mt protein-coding genes (*nad1, nad2, nad3, nad4*, and *nad5*) were excluded from our analysis because their sequences were not available for all of the species above. Protein-coding gene sequences were aligned based on amino acid sequences using the MAFFT algorithm implemented in TranslatorX online platform [32]. rRNA genes were aligned using the MAFFT v7.0 online server with G-INS-i strategy [33]. Individual gene alignments were concatenated after removing poorly aligned sites using GBlocks v0.91b [34]. A concatenated alignment, PCGRNA matrix, was used in subsequent analyses; this matrix combines the sequences of the eight protein-coding genes and the two rRNA genes (4,631 bp in total). The matrix was analyzed using maximum likelihood (ML) and Bayesian methods with MEGA-X [35] and 2) Bayesian inference method (BI) with MrBayes 3.2.6 [36], respectively. For ML analysis, the number of bootstrap replicates was 500; the substitution model selected was *Tamura-Nei* and the heuristic method selected was *Nearest-Neighbor-Interchange (NNI)*. For Bayesian analyses, four independent Markov chains were run for 25,000 MCMC generations, sampling a tree every 10 generations. Bayesian analysis was run until the average standard deviation of split frequencies was below 0.001. The initial 10,000 trees of each MCMC run were discarded as burn-in. ML trees were directly generated in MEGA-X and the Bayesian trees were drawn with Figtree v1.4.3.

**Table 1:** Species of parasitic lice included in the phylogenetic analyses in this study

Species	Family	Suborder	Order	GenBank accession number	References
*Polyplax reclinata*	Polyplacidae	Anoplura	Phthiraptera	MW291451-MW291461	Present study
*Hoplopleura akanezumi*	Hoplopleuridae	Anoplura	Phthiraptera	KJ648922–32	Dong et al., 2014a
*Hoplopleura kitti*	Hoplopleuridae	Anoplura	Phthiraptera	KJ648933–43	Dong et al., 2014a
*Polyplax spinulosa*	Polyplacidae	Anoplura	Phthiraptera	KF647762–72	Dong et al., 2014b
*Polyplax asiatica*	Polyplacidae	Anoplura	Phthiraptera	KF647751–61	Dong et al., 2014b
*Haematopinus asini*	Haematopinidae	Anoplura	Phthiraptera	KF939318, KF939322, KF939324, KF939326, KJ434034–38	Song et al., 2014
*Haematopinus apri*	Haematopinidae	Anoplura	Phthiraptera	KC814611–19	Jiang et al., 2013
*Haematopinus suis*	Haematopinidae	Anoplura	Phthiraptera	KC814602–10	Jiang et al., 2013
*Pthirus pubis*	Pthiridae	Anoplura	Phthiraptera	EU219988–95, HM241895–98	Shao et al., 2012
*Pediculus schaeffi*	Pediculidae	Anoplura	Phthiraptera	KC241882–97, KR706168–69	Herd et al., 2015
*Pediculus capitis*	Pediculidae	Anoplura	Phthiraptera	JX080388–407	Shao et al., 2012
*Pediculus humanus*	Pediculidae	Anoplura	Phthiraptera	FJ499473–90	Shao et al., 2009
*Pedicinus obtusus*	Pedicinidae	Anoplura	Phthiraptera	MT792495-506	Fu et al., 2020
*Pedicinus badii*	Pedicinidae	Anoplura	Phthiraptera	MT721726-39	Fu et al., 2020
*Microthoradus praelongiceps*	Microthoraciidae	Anoplura	Phthiraptera	KX090378–89	Shao et al., 2017
*Haematomyzus elephantis*	Haematomyzidae	Rhyncophthirina	Phthiraptera	KF933032–41	Shao et al., 2015

### Inference of the ancestral mitochondrial karyotype of ***Polyplax*** lice

**Fig. 1:**
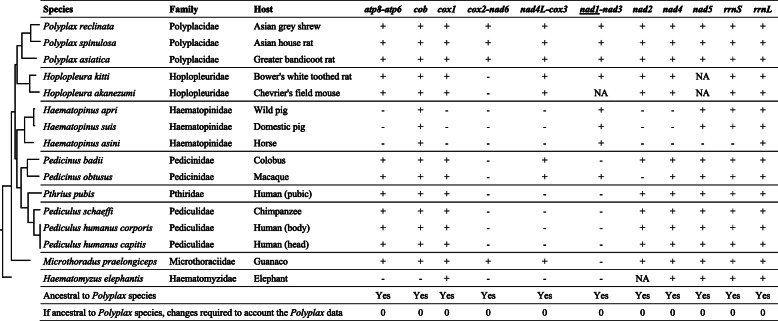
Inferring the ancestral mitochondrial karyotype of *Polyplax* lice (suborder Anoplura). tRNA genes are excluded. Plus symbol (+) is for presence, minus (-) for absence, and NA for information not available. Underlined *nad1* has an opposite orientation of transcription to other genes. The phylogenetic tree was inferred with mitochondrial genome sequences in the current study

**Fig. 2:**
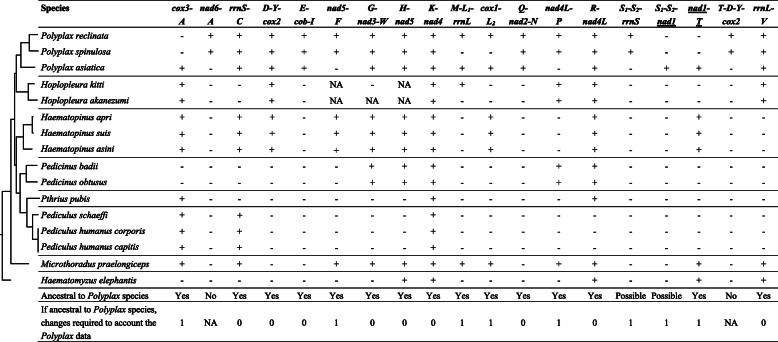
Inferring the location of tRNA genes relative to protein-coding and rRNA genes in the ancestral mitochondrial karyotype of *Polyplax* lice. tRNA genes are indicated with their single-letter abbreviations. Plus symbol (+) is for presence, minus (-) for absence, and NA for information not available. Underlined genes have opposite orientation of transcription to other genes. The phylogenetic tree was inferred with mitochondrial genome sequences in the current study

We used a parsimony method described in Shao et al., (2017) [9] to infer the ancestral mt karyotype of *Polyplax* lice based on information from the complete mt genomes of three *Polyplax* species: *P. reclinata* (present study), *P. asiatica* [4] and *P. spinulosa* [4]. We mapped characters of mt karyotypes on the phylogenetic tree of sucking lice inferred from mt genome sequences to identify shared characters. We inferred an mt minichromosome character to be ancestral to *Polyplax*: (1) if it was present in at least one *Polyplax* species and also in one or more non-*Polyplax* species of sucking lice; or (2) if it was present in all of the three *Polyplax* species. We considered tRNA genes separately from protein-coding and ribosome RNA genes because protein-coding and rRNA genes were much more stable than tRNA genes [9] (Figs. 1 and 2). For each inferred ancestral minichromosome character of *Polyplax* lice, we counted the changes needed to explain the observed mt karyotypes of *Polyplax* lice. If two or more minichromosome characters conflicted with one another, the character with the least required changes was inferred to be the ancestral. If two or more characters are equally parsimonious, all characters are inferred to be equally likely to be ancestral (Figs. 1 and 2).

## Results and discussion

### Mitochondrial genome of the Asian grey shrew louse, ***Polyplax reclinata***

**Fig. 3:**
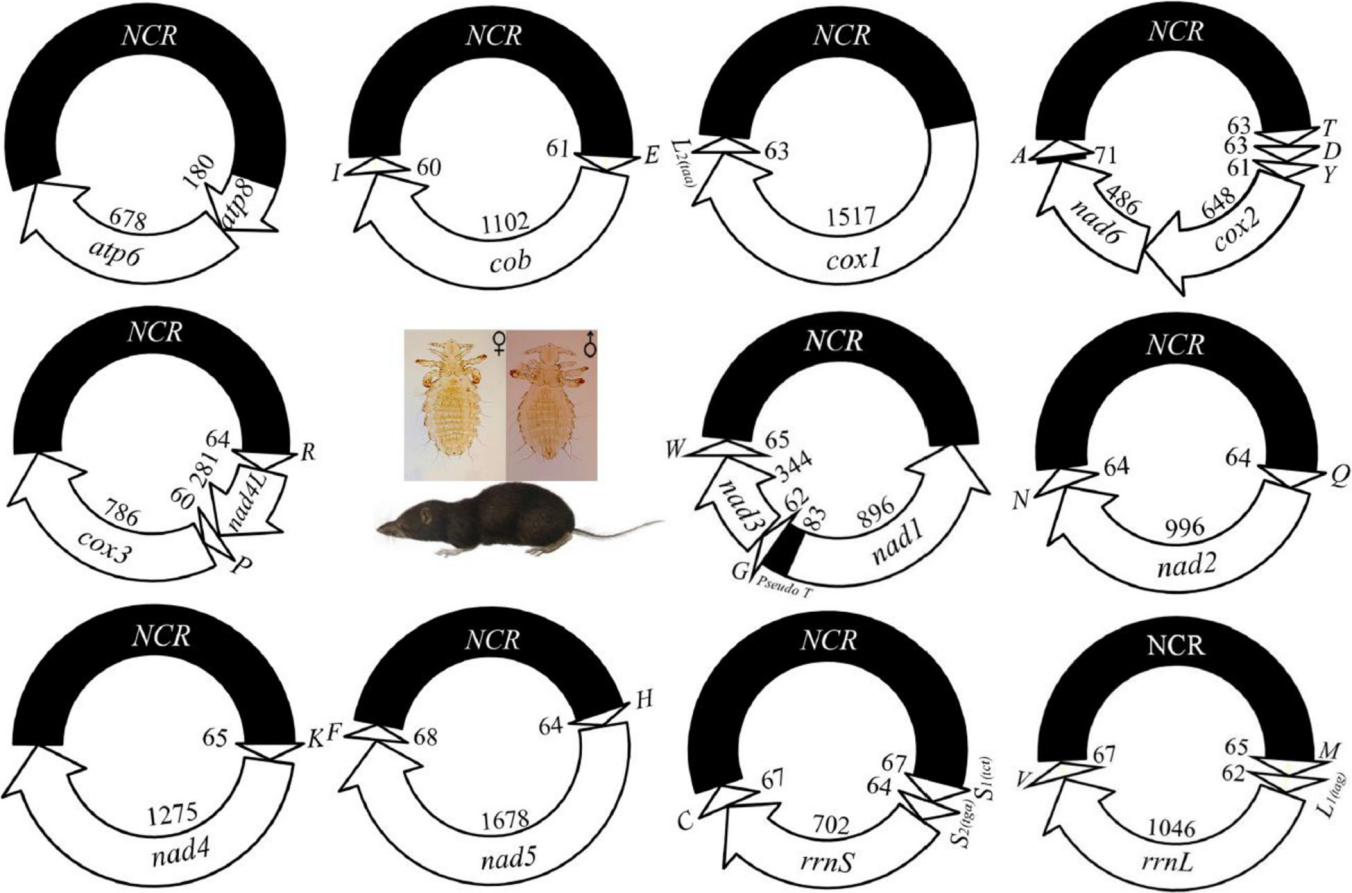
The mitochondrial genome of the Asian grey shrew louse, *Polyplax reclinata*. Protein-coding genes are: *atp6* and *atp8* (for ATP synthase subunits 6 and 8), *cox1*-*3* (for cytochrome c oxidase subunits 1–3), *cob* (for cytochrome b), *nad1*-*6* and *nad4L* (for NADH dehydrogenase subunits 1–6 and 4L). rRNA genes are: *rrnS* and *rrnL* (for small and large subunits of ribosomal RNA). tRNA genes are indicated with their single-letter abbreviations. Arrow indicates transcription orientation; numbers inside inner circles indicate gene length (bp); non-coding regions (*NCR*) are shaded in black

We obtained the complete mt genome of *P. reclinata* by assembling the 53,915,414 clean Illumina sequence reads (paired-end, 250 bp each) generated from the minichromosome amplicons (Table 2). All of the 37 mt genes typical of bilateral animals were identified in *P. reclinata*; these genes were on 11 circular minichromosomes (Fig. 3). The mt minichromosomes of *P. reclinata* range from 2,332 to 2,869 bp in size (Table 2); each minichromosome has a coding region and a non-coding region (Fig. 3; Table 2). The coding region of each minichromosome has 2–6 genes and ranges from 847 bp for *atp8*-*atp6* minichromosome to 1,809 bp for *H-nad5-F* minichromosome (minichromosomes named after the genes they contain). Seven of the 11 minichromosomes of *P. reclinata* contain a single protein-coding or rRNA gene each; the other four minichromosomes contain two protein-coding genes each. There are 1–4 tRNA genes in each minichromosome except *atp8-atp6* minichromosome, which has no tRNA genes (Fig. 3). Each of the 37 mt genes in *P. reclinata* is found only in one minichromosome; all genes have the same orientation of transcription relative to the non-coding region except for *nad1*, which has an opposite orientation to other genes (Fig. 3).

**Fig. 4:**
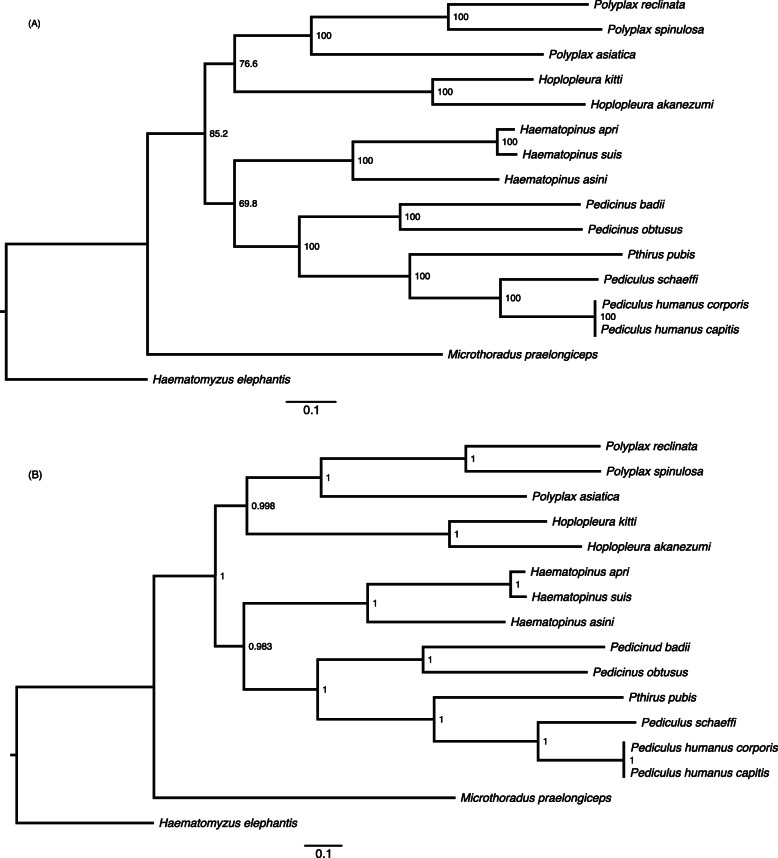
Phylogenetic relationships among 15 species of sucking lice (suborder Anoplura) inferred with combined sequences of eight mitochondrial protein-coding genes (*atp6*, *atp8, cox1*, *cox2*, *cox3, cob*, *nad4L*, *nad6*) and two rRNA genes (*rrnS* and *rrnL*) with maximum likelihood method (**A**) and Bayesian method (**B**). The trees were rooted with the elephant louse, *Haematomyzus elephantis* (suborder Rhyncophthirina). Bootstrap support values (out of 100) and Bayesian posterior probability values (out of 1) were indicated near internal nodes

**Table 2:** The mitochondrial minichromosomes of the Asian grey shrew louse, *Polyplax reclinata*, identified by Illumina sequencing

Minichromosome	Minichromosome size (bp)	Size of coding region (bp)	Size of non-coding region (bp)	Number of Illumina sequence reads	Mean coverage	GenBank accession number
*atp8-atp6*	2,425	847	1,578	5,836,284	333,785	MW291451
*E-cob-I*	2,620	1,223	1,397	7,275,348	387,778	MW291453
*cox1-L*_*2*_	2,816	1,580	1,236	4,012,234	204,899	MW291452
*T-D-Y-cox2-nad6-A*	2,473	1,399	1,074	3,691,415	212,776	MW291461
*R-nad4L-P-cox3*	2,465	1,191	1,274	6,293,311	365,198	MW291459
*nad1-G-nad3-W* ^a^	2,693	1,450	1,243	9,485,576	351,940	MW291457
*Q-nad2-N*	2,558	1,124	1,434	5,969,577	330,770	MW291458
*K-nad4*	2,680	1,340	1,340	4,248,148	218,975	MW291455
*H-nad5-F*	2,869	1,809	1,060	4,258,208	209,453	MW291454
*S*_*1*_*-S*_*2*_*-rrnS-C*	2,332	900	1,432	441,782	42,400	MW291460
*M-L*_*1*_*-rrnL-V*	2,559	1,240	1,319	2,403,531	213,096	MW291456
Total	28,490	14,103	14,387	53,915,414	2,871,070	

Note: ^a^underlined *nad1* is opposite to other genes in the orientation of transcription

We sequenced the non-coding regions of all of the 11 minichromosomes of *P. reclinata* in full length. The non-coding regions range from 1,060 to 1,578 bp (Table 2); the size variation is due to the additional sequences in the middle of the non-coding regions of *M-L*_*1*_*-rrnL-V*, *S*_*1*_*-S*_*2*_*-rrnS-C*, *K-nad4*, *atp8-atp6*, *Q-nad2-N* and *E-cob-I* minichromosomes (Additional file 4). If the additional sequences are excluded, the non-coding regions of all 11 minichromosomes have high sequence similarity to each other. Overall, the non-coding regions have high C and G content in one end but high A and T content in the other end. Indeed, a CG-rich motif (54 bp, 59 % C and G) was found immediately downstream from the 3’-end of the coding regions and an AT-rich motif (113 bp, 68 % A and T) was found upstream from the 5’-end of the coding regions in all of the minichromosomes (Additional file 4).

### Mitochondrial karyotype variation among ***Polyplax*** species

*Polyplax reclinata* differs in mt karyotype from the two other *Polyplax* species reported previously [4], although these three species all have 11 minichromosomes. *Polyplax reclinata* shares nine minichromosomes with *P. spinulosa* (louse of the Asian house rat) [4] but differs in the distribution of *trnL*_*1*_ and *trnL*_*2*_ between the other two minichromosomes (Table 3). In *P. spinulosa*, *trnL*_*1*_ is downstream from *cox1* and *trnL*_*2*_ is upstream from *rrnL* [4]; in *P. reclinata*, however, it is the opposite (Fig. 3; Table 3). *Polyplax reclinata* shares only four minichromosomes with *P. asiatica* (louse of the greater bandicoot rat) [4] and differs in the distribution of six tRNA genes among the other seven minichromosomes: *trnA*, *trnF*, *trnP*, *trnS*_*1*_, *trnS*_*2*_ and *trnT* (Table 3). If tRNA genes are excluded, the three *Polyplax* species are identical to one another in the distribution of protein-coding genes and rRNA genes among the 11 minichromosomes (Table 3; Fig. 3).

**Table 3:** Comparison of gene content and gene arrangement in the mitochondrial minichromosomes of three *Polyplax* species and their most recent common ancestor (MRCA)

Inferred most recent common ancestor of *Polyplax*	*Polyplax reclinata*	*Polyplax spinulosa*	*Polyplax asiatica*
*atp8-atp6*	*atp8-atp6*	*atp8-atp6*	*atp8-atp6*
*E-cob-I*	*E-cob-I*	*E-cob-I*	*E-cob-I*
*cox1-L*_*2(taa)*_	*cox1-L*_*2(taa)*_	*cox1-****L***_***1(tag)***_	*cox1-L*_*2(taa)*_
*D-Y-cox2-nad6*	***T****-D-Y-cox2-nad6-****A***	***T****-D-Y-cox2-nad6-****A***	*D-Y-cox2-nad6*
*R-nad4L-P-cox3-A*	*R-nad4L-P-cox3*	*R-nad4L-P-cox3*	*R-nad4L-cox3-A*
*S*_*1(tct)*_*-S*_*2(tga)*_*-nad1-T-G-nad3-W*	*nad1-G-nad3-W*	*nad1-G-nad3-W*	***S***_***1(tct)***_***-S***_***2(tga)***_*-nad1-T-G-nad3-W*
*Q-nad2-N*	*Q-nad2-N*	*Q-nad2-N*	*Q-nad2-N-****P***
*K-nad4*	*K-nad4*	*K-nad4*	*K-nad4-****F***
*H-nad5-F*	*H-nad5-F*	*H-nad5-F*	*H-nad5*
*S*_*1(tct)*_*-S*_*2(tga)*_*-rrnS-C*	***S***_***1(tct)***_***-S***_***2(tga)***_*-rrnS-C*	***S***_***1(tct)***_***-S***_***2(tga)***_*-rrnS-C*	*rrnS-C*
*M-L*_*1(tag)*_*-rrnL-V*	*M-L*_*1(tag)*_*-rrnL-V*	*M-****L***_***2(taa)***_*-rrnL-V*	*M-L*_*1(tag)*_*-rrnL-V*

Notes: The position of *S*_*1(tct)*_*-S*_*2(tga)*_ gene cluster in the ancestral mitochondrial karyotype is either upstream from *rrnS* or, equally parsimoniously, downstream from *nad1*. The eight tRNA genes that have changed their positions and/or orientation in the three *Polyplax* species since their divergence from their MRCA are in bold

### Phylogeny of sucking lice inferred from mt genome sequences

To assist the inference of the ancestral mt karyotype of *Polyplax* lice, we inferred the phylogeny of sucking lice using mt genome sequences of 15 species of sucking lice of seven genera including *Polyplax reclinata*, which was sequenced in the present study. The elephant louse, which was in the suborder Rhynchophthirina, was used as the outgroup (Table 1). Rhynchophthirina is the sister suborder to the sucking lice (suborder Anoplura) [10]. We obtained two trees from PCGRNA matrix (4,631 bp of protein-coding and rRNA gene sequences) with ML and Bayesian methods. These two trees have similar topologies and consistently support the monophyly of the genus *Polyplax* (Fig. 4): bootstrap support value (BSV) = 100 %, posterior probability (PP) = 1. Within the *Polyplax*, *P. reclinata* is more closely related to *P. spinulosa* than to *P. asiatica* (BSV = 100 %, PP = 1), despite the fact that the latter two species are found mostly on rodents whereas *P. reclinata* is found on shrews [37, 38]. The other four genera, *Hoplopleura*, *Pediculus*, *Pedicinus* and *Haematopinus*, also had multiple species from each genus included in our analysis; each of these genera was monophyletic with strong support (BSV = 100 %, PP = 1) (Fig. 4). The 15 species of sucking lice were divided into three clades: *Polyplax* and *Hoplopleura* forming a clade (BSV = 76.6 %: PP = 0.998); *Pediculus*, *Pedicinus, Pthirus* and *Haematopinus* in another clade (BSV = 69.8 %; PP = 0.983); and *Microthoracius praelongiceps* alone as the earliest branch (BSV = 85.2 %; PP = 1) (Fig. 4). Each of the genera included in our analysis also represents a family (Table 1). The family level relationships revealed by our analysis are consistent with that proposed by Kim (1988) based on morphological characters [39] and partially consistent with that by Light et al. (2010), Smith et al. (2011) and Johnson et al. (2018) based on gene sequences [13–15].

### Inferred ancestral mitochondrial karyotype of ***Polyplax*** lice

**Fig. 5:**
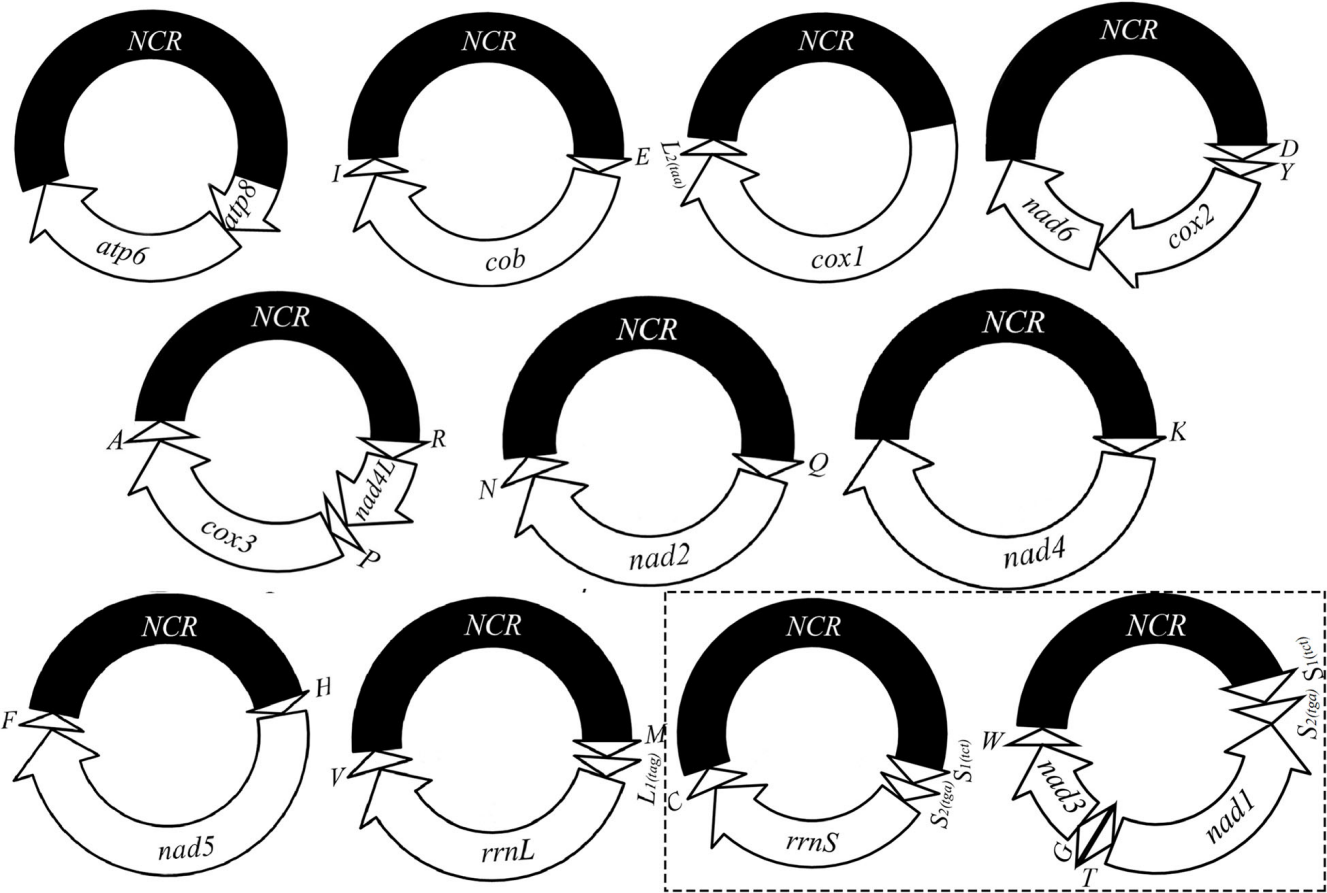
Inferred ancestral mitochondrial karyotype of *Polyplax* lice (suborder Anoplura). Abbreviated gene names are the same as in Fig. 3. Arrow indicates transcription orientation; non-coding regions (*NCR*) are shaded in black. The two minichromosomes containing *S*_*1*_*-S*_*2*_ gene cluster are boxed in dash line because the location of this cluster is either upstream from *rrnS* or, equally parsimoniously, downstream from *nad1*

We inferred the ancestral mt karyotype of *Polyplax* lice based on the data available for the three *Polyplax* species and the other 12 species of sucking lice (Table 1; Fig. 5). We mapped mt karyotype characters on the phylogeny inferred from the mt genome sequences of these lice to identify shared characters (Figs. 1 and 2). The inferred ancestral mt karyotype of *Polyplax* lice by parsimony consists of 11 minichromosomes; each minichromosome has a coding region with 2–7 genes and a non-coding region (Fig. 5). Seven of the 11 minichromosomes have a single protein-coding or rRNA gene each; the other four minichromosomes have two protein-coding genes each: (1) *atp6* and *atp8*, (2) *cox2* and *nad6*, (3) *cox3* and *nad4L*, and (4) *nad3* and *nad1* (Fig. 5). The position of all genes in the ancestral mt karyotype can be inferred without conflict except the position of a cluster of two tRNA genes, *S*_*1*_*-S*_*2*_, which is either upstream from *rrnS* or, equally parsimoniously, downstream from *nad1* (Figs. 2 and 5).

### Mitochondrial tRNA gene translocation in ***Polyplax*** lice

When compared with the inferred ancestral mt karyotype of *Polyplax* lice (Fig. 5), all three *Polyplax* species sequenced to date retained 11 minichromosomes and the same distribution pattern of protein-coding and rRNA genes among the minichromosomes (Table 3). Therefore, no split nor merger of minichromosomes have occurred in any of these *Polyplax* species since their most recent common ancestor (MRCA) (Fig. 4). However, these three *Polyplax* species are different from one another and from their MRCA in the position of tRNA genes. In total, eight tRNA genes have changed their positions in the three *Polyplax* lice since they diverged from their MRCA: *trnA*, *trnF*, *trnL*_*1*_, *trnL*_*2*_, *trnP*, *trnS*_*1*_, *trnS*_*2*_ and *trnT* (Table 3). *trnA* and *trnT* were likely translocated (*trnT* also inverted) in the MRCA of *P. reclinata* and *P. spinulosa* (Fig. 4) and thus are shared by these two species (Table 3). A pseudo *trnT* can be found in *P. reclinata* (Fig. 3) and *P. spinulosa* [4], indicating the translocation of *trnT* occurred relatively recently. *trnL*_*1*_ and *trnL*_*2*_ swapped their positions in *P. spinulosa* [4], most likely caused by point mutations at the third anti-codon position (or by homologous recombination between *trnL*_*1*_ and *trnL*_*2*_) – a mechanism suggested by previous studies [2, 9]. *trnF* and *trnP* were translocated in *P. asiatica* (Table 3); as a cluster, *trnS*_*1*_-*trnS*_*2*_ was translocated either in *P. asiatica* or equally likely in the MRCA of *P. reclinata* and *P. spinulosa* (Table 3; Fig. 5).

### tRNA gene translocation is frequent and directional towards the boundaries with control region in parasitic lice with fragmented mitochondrial genomes

We noted in the *Polyplax* lice above that mt tRNA gene translocation was closely associated with the control region, i.e. the non-coding region (NCR). With no exception, the destination of all of the translocated tRNA genes (i.e. *trnA*, *trnF*, *trnS*_*1*_-*trnS*_*2*_, *trnP* and *trnT* excluding *trnL*_*1*_ and *trnL*_*2*_ which swapped positions in *P. spinulosa* as discussed above) were either immediately next to the NCR or in the case of *trnP* of *P. asiatica*, a short distance (189 bp) inside the NCR (Table 3) [4], indicating a possible role of NCR in tRNA gene translocation. The observed probability of tRNA gene translocation to be adjacent to NCR in these *Polyplax* species is significantly higher (*p* < 0.00001) than the expected probability (Table 4).

**Table 4:** Statistical analysis of expected probability and observed probability of tRNA gene translocation in three *Polyplax* species relative to their most recent common ancestor

tRNA genes	Expected probability of translocation to be adjacent to NCR if translocation is random	Observed probability of translocation to be adjacent to NCR	Expected probability of translocation to an intergenic region if translocation is random	Observed probability of translocation to an intergenic region
*A*	43.75 % (21/48)	100 % (2/2)	56.25 % (27/48)	0 %
*F*	43.75 % (21/48)	100 % (1/1)	56.25 % (27/48)	0 %
*P*	45.83 % (22/48)	100 % (1/1)	54.12 % (26/48)	0 %
*T*	45.83 % (22/48)	100 % (2/2)	54.12 % (26/48)	0 %
*S*_*1*_*-S*_*2*_	44.68 % (21/47)	100 % (3/3)	55.32 % (26/47)	0 %
Average	44.8 %	100 %	55.2 %	0 %
Two tailed *t*-value	-118.61978		115.73698	
*p*-value	< 0.00001		< 0.00001	
Significant at *p* < 0.01?	Yes		Yes	

Notes: Refer to Table 3 for tRNA gene translocation in *Polyplax* species. “Expected probability of translocation to be adjacent to NCR” = “total sites adjacent to NCR” / “total sites adjacent to NCR + total intergenic sites”. “Expected probability of translocation to an intergenic region” = “total intergenic sites” / “total sites adjacent to NCR + total intergenic sites”. “Observed probability of translocation to be adjacent to NCR” = “translocation adjacent to NCR” / “translocation adjacent to NCR + translocation to intergenic sites”. “Observed probability of translocation to an intergenic region” = “translocation to intergenic sites” / “translocation adjacent to NCR + translocation to intergenic sites”. *T*-Test was done at: https://www.socscistatistics.com/tests/studentttest/default2.aspx

We investigated further if this was a broader pattern for parasitic lice with fragmented mt genomes by comparing the mt karyotypes: (1) between the MRCA of sucking lice [9] and the MRCA of *Polyplax* (Fig. 5); and (2) between the MRCA of sucking lice and each of the 12 non-*Polyplax* sucking louse species that have been sequenced to date [9, 12]. The MRCA of *Polyplax* species retained the same distribution pattern of protein-coding and rRNA genes and the same number of minichromosomes as the MRCA of sucking lice. However, five tRNA genes have translocated in the MRCA of *Polyplax* relative to the MRCA of sucking lice: all of these tRNA genes were translocated immediately next to the NCR despite alternative intergenic locations available for translocation (Table 5). The observed probability of tRNA gene translocation to be adjacent to NCR in the MRCA of *Polyplax* species is significantly higher (*p* < 0.00001) than the expected probability (Table 6). When compared with the MRCA of sucking lice, 19 of the 22 tRNA genes in total have translocated in the 12 species of non-*Polyplax* sucking lice: 32 out of the 54 (i.e. 59 %) translocation events resulted in tRNA genes moved to be adjacent to NCR despite alternative intergenic locations available for translocation (Table 7). The observed probability of tRNA gene translocation to be adjacent to NCR in these non-*Polyplax* species is 13.6 % higher than the expected probability but this difference is not statistically significant (*p* = 0.116709) (Table 8). Most likely tRNA gene translocation to be adjacent to NCR has been under-captured in non-*Polyplax* species (Table 7) due to the fact that any newly translocated tRNA gene adjacent to a NCR would inevitably push the previously translocated tRNA gene away from the NCR. After all, there can only be one gene at any time adjacent to each end of the NCR. Among the three comparisons above, the third comparison has the longest evolutionary time frame. The longer the evolutionary time is, the more likely tRNA gene translocation information to be adjacent to NCR will be under-captured.

**Table 5:** Translocated tRNA genes in the most recent common ancestor (MRCA) of *Polyplax* lice relative to the MRCA of sucking lice

tRNA gene	MRCA of sucking lice	MRCA of *Polyplax*	Translocated	Next to NCR
*N*	*atp8-atp6-N*	*Q-nad2-****N***	Yes	Yes
*S*_*1*_*-S*_*2*_	*E-cob-S*_*1*_*-S*_*2*_	***S***_***1***_***-S***_***2***_*-rrnS-C* *or* ***S***_***1***_***-S***_***2***_*-nad1-T-G-nad3-W*	Yes	Yes
*I*	*I-cox1-L*_*2*_	*E-cob-****I***	Yes	Yes
*Q*	*Q-nad1-T-nad1-G-nad3-W*	***Q****-nad2-N*	Yes	Yes

Note: translocated tRNA genes in the MRCA of *Polyplax* lice are in bold

**Table 6:** Statistical analysis of expected probability and observed probability of tRNA gene translocation in the most recent common ancestor (MRCA) of *Polyplax* lice relative to the MRCA of sucking lice

tRNA genes	Expected probability of translocation to be adjacent to NCR if translocation is random	Observed probability of translocation to be adjacent to NCR	Expected probability of translocation to an intergenic region if translocation is random	Observed probability of translocation to an intergenic region
*N*	21/46 = 45.65 %	100 %	25/46 = 54.35 %	0 %
*S*_*1*_*-S*_*2*_	21/45 = 46.67 %	100 %	24/45 = 53.33 %	0 %
*I*	21/46 = 45.65 %	100 %	25/46 = 54.35 %	0 %
*Q*	21/46 = 45.65 %	100 %	25/46 = 54.35 %	0 %
Average	45.9 %	100 %	54.1 %	0 %
Two tailed *t*-value	-212.13726		212.13726	
*p*-value	< 0.00001		< 0.00001	
Significant at *p* < 0.01?	Yes		Yes	

Notes: Refer to Table 5 for tRNA gene translocation in the MRCA of *Polyplax* species. Refer to Table 4 notes for probability calculation and *T*-Test

**Table 7:** Translocated tRNA genes in non-*Polyplax* sucking lice relative to the most recent common ancestor (MRCA) of sucking lice

tRNA gene	MRCA of sucking lice	Non-*Polyplax* sucking lice	Species	Translocated	Next to NCR
*N*	*atp8-atp6-N*	*E-cob-S*_*1*_*-S*_*2*_*-****N***	*Microthoracius praelongiceps*[9]	Yes	Yes
		*cob-S*_*1*_*-****N****-E-M*	*Pediculus schaeffi*[7]	Yes	No
		*S*_*1*_*-****N****-E*	*Pediculus capitis*[2]	Yes	No
			*Pediculus humanus*[2]		
		*T-nad1-Q-****N****-C*	*Pedicinus badii*[12]	Yes	No
		*T-nad1-Q-****N****-G-nad3-W*	*Pedicinus obtusus*[12]	Yes	No
*E*	*E-cob-S*_*1*_*-S*_*2*_	*cob-S*_*1*_*-N-****E****-M*	*Pediculus schaeffi*[7]	Yes	No
		*F-nad6-****E****-M*	*Pthirus pubis*[2]	Yes	No
		*S*_*1*_*-N-****E***	*Pediculus capitis*[2]	Yes	Yes
			*Pediculus humanus*[2]		
*S*_*1*_	*E-cob-S*_*1*_*-S*_*2*_	*D-Y-cox2-****S***_***1***_*-S*_*2*_*-P-cox3-A*	*Haematopinus apri*[3]	Yes	No
			*Haematopinus suis*[3]		
			*Haematopinus asini*[6]		
		***S***_***1***_*-N-E*	*Pediculus capitis*[2]	Yes	Yes
			*Pediculus humanus*[2]		
*S*_*2*_	*E-cob-S*_*1*_*-S*_*2*_	*D-Y-cox2-S*_*1*_*-****S***_***2***_*-P-cox3-A*	*Haematopinus apri*[3]	Yes	No
			*Haematopinus suis*[3]		
			*Haematopinus asini*[6]		
		*G-nad3-V-W-****S***_***2***_	*Pthirus pubis*[2]	Yes	Yes
*I*	*I-cox1-L*_*2*_	*P-nad2-****I***	*Pediculus schaeffi*[7]	Yes	Yes
			*Pediculus capitis*[2]		
			*Pediculus humanus*[2]		
			*Pthirus pubis*[2]		
		*R-nad4L-P-cox3-****I***	*Pedicinus obtusus*[12]	Yes	Yes
*L*_*2*_	*I-cox1-L*_*2*_	***L***_***2***_*-rrnS-C*	*Pediculus schaeffi*[7]	Yes	Yes
			*Pediculus capitis*[2]		
			*Pediculus humanus*[2]		
*D*	*D-Y-cox2-nad6*	*T-****D****-H-R-nad4L*	*Pthirus pubis*[2]	Yes	No
		*T-****D****-H*	*Pediculus schaeffi*[7]	Yes	No
			*Pediculus capitis*[2]		
			*Pediculus humanus*[2]		
		*G-nad3-W-****D****-A-V*	*Pedicinus badii*[12]	Yes	No
		***D****-A-M-F-nad6*	*Pedicinus obtusus*[12]	Yes	Yes
*P*	*R-nad4L-P-cox3-A*	***P****-nad2-I*	*Pthirus pubis*[2]	Yes	Yes
			*Pediculus schaeffi*[7]		
			*Pediculus capitis*[2]		
			*Pediculus humanus*[2]		
*A*	*R-nad4L-P-cox3-A*	*G-nad3-W-D-****A****-V*	*Pedicinus badii*[12]	Yes	No
		*D-****A****-M-F-nad6*	*Pedicinus obtusus*[12]	Yes	No
*Q*	*Q-nad1-T-G-nad3-W*	***Q****-N-E*	*Pediculus humanus*[2]	Yes	Yes
		*T-nad1-****Q****-N-C*	*Pedicinus badii*[12]	Yes	No
		*T-nad1-****Q****-N-G-nad3-W*	*Pedicinus obtusus*[12]	Yes	No
*T*	*Q-nad1-T-G-nad3-W*	***T****-nad1-Q-N-C*	*Pedicinus badii*[12]	Yes	Yes
		***T****-nad1-Q-N-G-nad3-W*	*Pedicinus obtusus*[12]	Yes	Yes
		*D-Y-cox2-****T***	*Hoplopleura kitti*[5]	Yes	Yes
		*R-nad4L-P-cox3-A-****T***	*Hoplopleura akanezumi*[5]	Yes	Yes
		***T****-D-H-R-nad4L*	*Pthirus pubis*[2]	Yes	Yes
		***T****-D-H*	*Pediculus schaeffi*[7]	Yes	Yes
			*Pediculus capitis*[2]		
			*Pediculus humanus*[2]		
		***T****-nad1-Q-N-C*	*Pedicinus badii*[12]	Yes	Yes
		***T****-nad1-Q-N-G-nad3-W*	*Pedicinus obtusus*[12]	Yes	Yes
*G*	*Q-nad1-T-G-nad3-W*	***G****-nad4L-V*	*Pediculus schaeffi*[7]	Yes	Yes
			*Pediculus capitis*[2]		
			*Pediculus capitis*[2]		
*W*	*Q-nad1-T-G-nad3-W*	*C-nad6-****W****-L*_*2*_	*Hoplopleura kitti*[5]	Yes	No
			*Hoplopleura akanezumi*[5]		
		*G-nad3-V-****W****-S*_*2*_	*Pthirus pubis*[2]	Yes	No
*H*	*H-nad5-F*	*T-D-****H****-R-nad4L*	*Pthirus pubis*[2]	Yes	No
		*T-D-****H***	*Pediculus schaeffi*[7]	Yes	Yes
			*Pediculus capitis*[2]		
			*Pediculus humanus*[2]		
*F*	*H-nad5-F*	***F****-nad6-E-M*	*Pthirus pubis*[2]	Yes	Yes
		*M-****F****-nad6*	*Pedicinus badii*[12]	Yes	No
		*D-A-M-****F****-nad6*	*Pedicinus obtusus*[12]	Yes	No
*C*	*rrnS-C*	***C****-nad6-W-L*_*2*_	*Hoplopleura kitti*[5]	Yes	Yes
			*Hoplopleura akanezumi*[5]		
		*T-nad1-Q-N-****C***	*Pedicinus badii*[12]	Yes	Yes
		*atp8-atp6-****C***	*Pedicinus obtusus*[12]	Yes	Yes
*M*	*M-L*_*1*_*-rrnL-V*	*R-nad4L-nad6-****M***	*Haematopinus apri*[3]	Yes	Yes
			*Haematopinus suis*[3]		
		*F-nad6-E-****M***	*Pthirus pubis*[2]	Yes	Yes
		*cob-S*_*1*_*-N-E-****M***	*Pediculus schaeffi*[7]	Yes	Yes
		***M****-F-nad6*	*Pedicinus badii*[12]	Yes	Yes
		*D-A-****M****-F-nad6*	*Pedicinus obtusus*[12]	Yes	No
*L*_*1*_	*M-L*_*1*_*-rrnL-V*	***L***_***1***_*-rrnS-C*	*Pediculus schaeffi*[7]	Yes	Yes
			*Pediculus capitis*[2]		
			*Pediculus humanus*[2]		
*V*	*M-L*_*1*_*-rrnL-V*	*G-nad3-****V****-W-S*_*2*_	*Pthirus pubis*[2]	Yes	No
		*G-nad3-W-D-A-****V***	*Pedicinus badii*[12]	Yes	Yes
		*nad2-Y-cox2-****V***	*Pedicinus obtusus*[12]	Yes	Yes
		*G-nad4L-****V***	*Pediculus schaeffi*[7]	Yes	Yes
			*Pediculus capitis*[2]		
			*Pediculus humanus*[2]		

Notes: Translocation events shared by congeneric species are grouped together and counted as single events. Genes underlined have opposite orientation of transcription to other genes

**Table 8:** Statistical analysis of expected probability and observed probability of tRNA gene translocation in non-*Polyplax* sucking lice relative to the most recent common ancestor of sucking lice

tRNA genes	Expected probability of translocation to be adjacent to NCR if translocation is random	Observed probability of translocation to be adjacent to NCR	Expected probability of translocation to an intergenic region if translocation is random	Observed probability of translocation to an intergenic region
*N*	21/45 = 46.67 %	20 %	24/45=53.33%	80 %
*E*	21/45 = 46.67 %	33.3 %	24/45 = 53.33 %	66.7 %
*S*_*1*_	22/46 = 47.83 %	50 %	23/46 = 52.17 %	50 %
*S*_*2*_	21/45 = 46.67 %	50 %	24/45 = 53.33 %	50 %
*I*	21/45 = 46.67 %	100 %	24/45 = 53.33 %	0 %
*L*_*2*_	21/45 = 46.67 %	100 %	24/45 = 53.33 %	0 %
*D*	21/45 = 46.67 %	25 %	24/45 = 53.33 %	75 %
*P*	22/46 = 47.83 %	100 %	23/46 = 52.17 %	0 %
*A*	21/45 = 46.67 %	0 %	24/45 = 53.33 %	100 %
*Q*	21/45 = 46.67 %	33.3 %	24/45 = 53.33 %	66.7 %
*T*	22/46 = 47.83 %	100 %	23/46 = 52.17 %	0 %
*G*	22/46 = 47.83 %	100 %	23/46 = 52.17 %	0 %
*W*	21/45 = 46.67 %	0 %	24/45 = 53.33 %	100 %
*H*	21/45 = 46.67 %	50 %	24/45 = 53.33 %	50 %
*F*	21/45 = 46.67 %	33.3 %	24/45 = 53.33 %	66.7 %
*C*	21/45 = 46.67 %	100 %	24/45 = 53.33 %	0 %
*M*	21/45 = 46.67 %	80 %	24/45 = 53.33 %	20 %
*L*_*1*_	22/46 = 47.83 %	100 %	23/46 = 52.17 %	0 %
*V*	21/45 = 46.67 %	75 %	24/45 = 53.33 %	25 %
Average	46.9 %	60.5 %	53.1 %	39.5 %
Two tailed *t*-value	-1.60738		1.61464	
*p*-value	0.116709		0.11512	
Significant at *p* < 0.01?	No		No	

Notes: Refer to Table 7 for tRNA gene translocation in non-*Polyplax* sucking lice. Refer to Table 4 notes for probability calculation and *T*-Test

Clearly, the boundaries between coding regions and NCRs are hot spots for tRNA gene translocation in parasitic lice that have fragmented mt genomes. Of the models and mechanisms proposed in previous studies (introduced above in the Background) [2, 4, 6, 9, 10], only the inter-minichromosomal recombination model proposed by Song et al., [6] based on the mt karyotype variation among *Haematopinus* lice can explain tRNA gene translocation between mt minichromosomes. However, it cannot explain why translocated tRNA genes tend to be in the boundaries between coding regions and NCRs. As DNA double-strand breaks (DSBs) are apparently necessary for gene translocation between mt minichromosomes, the boundary regions may have more frequent DSBs than other regions of the mt minichromosomes, possibly due to the specific AT-rich and GC-rich sequence elements in the boundary regions; these sequence elements are conserved among all mt minichromosomes of each species of parasitic louse (Additional file 4). Apparently, DSBs do not occur randomly. Tubbs et al. showed that in mouse and human cells, DSBs occur mostly in AT-rich regions, in particular at homopolymeric (dA/dT) tracts [40]. Sinai et al. showed that integration of AT-dinucleotide rich sequences into human chromosomes created recurrent gaps and breaks at the integration sites, due to a significantly increased tendency to fold into branched secondary structures at these sites [41]. Baudat et al. showed that a histone methyl transferase, PRDM9, is a major determinant of meiotic recombination hotspots in humans and mice, and furthermore, the human PRDM9 binding sites contain a 13-mer GC-rich motif and are DSB hotspots [42]. Sundararajan et al. showed that DSB hotspots have a strong tendency to occur in genes with high GC content in maize [43]. He et al. investigated the DNA sequence of DSB hotspots and identified a 20-bp-long GC-rich degenerate DNA sequence motif (named Maize Hotspot Sequence) present in about 72 % of genic hotspots in maize [44]. In general, non-canonical DNA structures such as hairpins and G-quadruplexes render a genome prone to damage including DSBs [45]. We counted the number of translocated tRNA genes in sucking lice at each end of the mt non-coding regions (Tables 3, 5 and 7) – the distribution is rather even (20 vs. 20) between the two ends where the AT-rich and GC-rich motifs were found respectively. Our observed pattern of mt tRNA gene translocation in sucking lice aligns well with the evidence presented in these experimental studies on DSB hotspots. Future studies on DSBs and DNA repair in parasitic lice may help understand further the mechanisms of mt tRNA gene translocation observed in sucking lice in the present study.

In conclusion, we sequenced the fragmented mt genome of the Asian grey shrew louse, *Polyplax reclinata*, which comprises 11 circular minichromosomes. We also inferred the ancestral mt karyotype of *Polyplax* lice based on the data available to date. We found that tRNA genes are entirely responsible for mt karyotype variation among the three species of *Polyplax* lice sequenced to date. Furthermore, tRNA gene translocation in these *Polyplax* lice is much more frequent than that of protein-coding and rRNA genes; all tRNA gene translocation observed occurs between different types of minichromosomes and is directional towards the boundaries with the control region. A similar pattern of tRNA gene translocation is also conserved in other sucking lice with fragmented mt genomes. Our results show that inter-minichromosomal tRNA gene translocation orientated towards the two ends of the control region is a major contributing factor to the highly dynamic mitochondrial genome organization in the parasitic lice of mammals.

## Supplementary Information

**Additional file 1:** Image of the voucher specimens (# 364) of the shrew louse, *Polyplax reclinata*.

**Additional file 2: **The primers used to amplify the mitochondrial genes, minichromosomes and coding regions of the Asian grey shrew louse, *Polyplax reclinata*.

**Additional file 3: **PCR amplicons generated from the mitochondrial (mt) minichromosomes of the Asian grey shrew louse, *Polyplax reclinata*. (A) Lane 1: amplicon of *S*_*1*_*-S*_*2*_*-rrnS-C* minichromosome generated with the primer pair 12S364F-12S364R. Lane 2: amplicon of *M-L*_*1*_*-rrnL-V* minichromosome generated with the primer pair 16S364F-16S364R. Lane 3: 500 bp Ladder (Tiangen) (band size in bp indicated). Lane 4: amplicons of coding regions of all mt minichromosomes generated with the primer pair 364 F-364R. Lane 5: GeneRuler 100 bp DNA Ladder (Thermo Scientific) (band size in bp indicated). The lane to the right of lane 2 is irrelevant to this manuscript. The lane to the left of lane 3 is empty. (B) Lane 1: 1 kb ladder (Tiangen) (band size in bp indicated); Lanes 2–10: amplicons of individual mt minichromosomes, *T-D-Y-cox2-nad6-A*, *R-nad4L-P-cox3*, *Q-nad2-N*, nad1 *-G-nad3-W* (gene underlined has opposite transcription orientation to other genes), *K-nad4*, *H-nad5-F*, *E-cob-I*, *cox1-L*_*2*_, *atp8-atp6*. Details of the primers used to amplify individual mt minichromosomes are provided in Additional file 2.

**Additional file 4:** Alignment of the non-coding region (*NCR*) sequences of 11 mitochondrial minichromosomes of the Asian grey shrew louse, *Polyplax reclinata*. The primer pair, 364 F and 364R, were used to amplify the coding regions of the 11 minichromosomes (see also Additional file 3). Asterisk symbol “*” indicates conserved nucleotides; hyphen “-” indicates absent nucleotides.

## Data Availability

The nucleotide sequence of the mitochondrial genome of the Asian grey shrew louse, *Polyplax reclinata*, has been deposited in GenBank (accession number MW291451-MW291461) and will be released publicly once this manuscript is accepted for publication.

## Abbreviations

µl: Microliter
*atp6* and *atp8*: Genes for ATP synthase subunits 6 and 8
bp: Base pair
*cob*: Gene for cytochrome b
*cox1*, *cox2* and * cox3*: Genes for cytochrome c oxidase subunits 1, 2 and 3
DNA: Deoxyribonucleic acid
kb: Kilo base pair
min: Minute
MRCA: Most recent common ancestor
mt: Mitochondrial
Mya: Million years ago
*nad1*, *nad2*, *nad3*, *nad4*, *nad4L*, *nad5* and * nad6*: Mitochondrial genes for NADH dehydrogenase subunits 1–6 and 4L
PCR: Polymerase chain reaction
RNA: Ribonucleic acid
rRNA: Ribosomal RNA
*rrnS * and *rrnL*: Genes for small and large subunits of ribosomal RNA
sec: Second
T: Thymine
tRNA: Transfer RNA
*trnA* or *A*: tRNA gene for alanine
*trnC* or * C*: tRNA gene for cysteine
*trnD* or * D*: tRNA gene for aspartic acid
*trnE * or * E*: tRNA gene for glutamic acid
*trnF or F*: tRNA gene for phenylalanine
*trnG or G*: tRNA gene for glycine
*trnH* or *H*: tRNA gene for histidine
*trnI* or * I*: tRNA gene for isoleucine
*trnK* or * K*: tRNA gene for lysine
*trnL*_*1*_ or * L*_*1*_: tRNA gene for leucine (anticodon NAG)
*trnL*_*2*_ or *L*_*2*_: tRNA gene for leucine (anticodon YAA)
*trnM* or *M*: tRNA gene for methionine
*trnN* or *N*: tRNA gene for asparagine
*trnP* or * P*: tRNA gene for proline
*trnQ* or * Q*: tRNA gene for glutamine
*trnR* or *R*: tRNA gene for arginine
*trnS*_*1 *_ or *S*_*1*_: tRNA gene for serine (anticodon NCU)
*trnS*_*2 *_ or * S*_*2*_: tRNA gene for serine (anticodon NGA)
*trnT* or * T*: tRNA gene for threonine
*trnV* or *V*: tRNA gene for valine
*trnW * or *W*: tRNA gene for tryptophan
*trnY* or * Y*: tRNA gene for tyrosine
U: Uracil
