# Supplementary material for: Frequent tRNA gene translocation towards the boundaries with control regions contributes to the highly dynamic mitochondrial genome organization of the parasitic lice of mammals
Source: BMC Genomics. 2021 Aug 6;22:598. doi: 10.1186/s12864-021-07859-w (PMC8344215; doi:10.1186/s12864-021-07859-w)

Research Article (Supplementary material)

**Frequent tRNA gene translocation towards the boundaries with control regions contributes to the highly dynamic mitochondrial genome organization of the parasitic lice of mammals**

Wen-Ge Dong^1*^, Yalun Dong^2,3^, Xian-Guo Guo^1^, Renfu Shao^2,3*^

^1^Institute of Pathogens and Vectors, Dali University, and the Key Laboratory for Preventing and Controlling Plague in Yunnan Province, Dali 671000, China

^2^GeneCology Research Centre, University of the Sunshine Coast, Maroochydore, Queensland, Australia

^3^School of Science, Technology and Engineering, University of the Sunshine Coast, Maroochydore, Queensland, Australia

^*^Corresponding authors: Wen-Ge Dong, [dongwenge2740@sina.com](mailto:dongwenge2740@sina.com); Renfu Shao, [rshao@usc.edu.au](mailto:rshao@usc.edu.au)

**Additional file 1.** Image of the voucher specimens (# 364) of the shrew louse, *Polyplax reclinata*


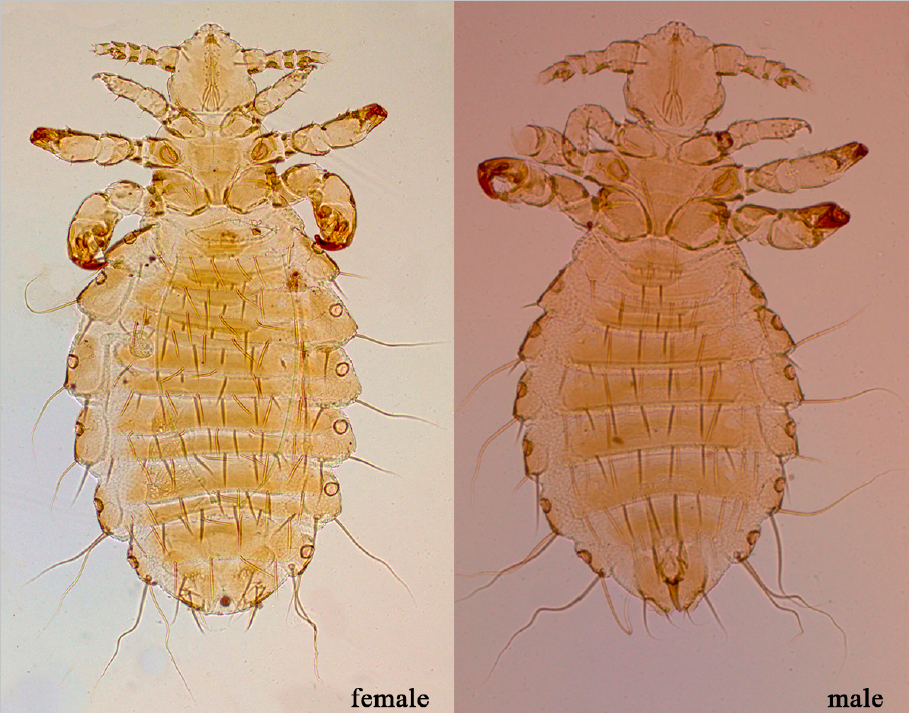

Supplement: Supplementary file 1 — Additional file 1: Image of the voucher specimens (# 364) of the shrew louse, Polyplax reclinata. [file 12864_2021_7859_MOESM1_ESM.docx]
