# Supplementary material for: Frequent tRNA gene translocation towards the boundaries with control regions contributes to the highly dynamic mitochondrial genome organization of the parasitic lice of mammals
Source: BMC Genomics. 2021 Aug 6;22:598. doi: 10.1186/s12864-021-07859-w (PMC8344215; doi:10.1186/s12864-021-07859-w)
Supplement: Supplementary file 2 — Additional file 2: The primers used to amplify the mitochondrial genes, minichromosomes and coding regions of the Asian grey shrew louse, Polyplax reclinata. [file 12864_2021_7859_MOESM2_ESM.docx]

**Additional file 2.** The primers used to amplify the mitochondrial genes, minichromosomes and coding regions of the Asian grey shrew louse, *Polyplax reclinata*

| **Primer** | **Target gene, region or minichromosome** | **Sequence** |
| --- | --- | --- |
| 12SA | *rrnS* gene | TACTATGTTACGACTTAT |
| 12SB | *rrnS* gene | AAACTAGGATTAGATACCC |
| 16SF | *rrnL* gene | TTAATTCAACATCGAGGTCGCAA |
| Lx16SR | *rrnL* gene | GACTGTGCTAAGGTAGCATAAT |
| 12S364F | *S_1_-S_2_-rrnS-C* minichromosome | ATGTCTTGTACATCGCTGTTTAGACACACC |
| 12S364R | *S_1_-S_2_-rrnS-C* minichromosome | CTCGTGCAGTTTCGTTTATGAACATATGCC |
| 16S364F | *M-L_1_-rrnL-V* minichromosome | AAGAAGGCTTAGAGTTACCTGGGAGGGGGC |
| 16S364R | *M-L_1_-rrnL-V* minichromosome | GGGTCTTCTCGTCCCTCTATGGCATTTGAGC |
| 364F | Entire coding regions of all minichromosomes | CAGGGTATAGAGGGCGCTCTGGATTGTAAG |
| 364R | Entire coding regions of all minichromosomes | CTTTCCCCCCCCAAAAAGGAGTCAGAGAC |
| 364atp6F | *atp8-atp6* minichromosome | GCTCCTATTGGACTTTCACCTTTTTTGGTG |
| 364atp6R | *atp8-atp6* minichromosome | GTTGGAAGGAAGTGAGCTAAAGTTCACTC |
| 364cobF | *E-cob-I* minichromosome | GTTACCATGAGGTCAGATGTCCTTTTGAGG |
| 364cobR | *E-cob-I* minichromosome | GTACCCGAGGAAAGCAGTAGCTATTAAAAC |
| 364cox1F | *cox1-L_2_* minichromosome | GTGGATAGACGAGCCTACTTTACTAGAGC |
| 364cox1R | *cox1-L_2_* minichromosome | CCCAACAGTAAATATATGGTGTGCCCACAC |
| 364cox2F | *T-D-Y-cox2-nad6-A* minichromosome | GAAGAAGTGGTCTTTCCTGAAATCAGGTC |
| 364cox2R | *T-D-Y-cox2-nad6-A* minichromosome | GAGACAATGCAAAGAAGGGAATGCTAAAGC |
| 364cox3F | *R-nad4L-P-cox3* minichromosome | GCCCTTCGCTTTAGTTGAAGACATTTTTCC |
| 364cox3R | *R-nad4L-P-cox3* minichromosome | GATTGTTCCCACAATCACATGAAGTCCGTG |
| 364nad1F | *nad1-G-nad3-W* minichromosome | CCCAAACGGTAGTACTGATAAGAACAAAGC |
| 364nad1R | *nad1-G-nad3-W* minichromosome | CAGACTCCTAACTCCTCAGGAGTAATTGC |
| 364nad2F | *Q-nad2-N* minichromosome | CTGTGAGTCATTCTTGGGATGAAAGTCAG |
| 364nad2R | *Q-nad2-N* minichromosome | GCAGAATATACTAAAGCCCGTCGAATAGAC |
| 364nad4F | *K-nad4* minichromosome | CTCATTATGAGGGAGGTTTGTATCTGCTC |
| 364nad4R | *K-nad4* minichromosome | GGCAAACTAAAAAGTCCCAAAATGCCGAC |
| 364nad5F | *H-nad5-F* minichromosome | GCTTTGTGAAGAGTTAGGGCAGATAGGTC |
| 364nad5R | *H-nad5-F* minichromosome | CTCGATTGACCCTGAGAAGGAGAATAGAAG |
