# Supplementary material for: Frequent tRNA gene translocation towards the boundaries with control regions contributes to the highly dynamic mitochondrial genome organization of the parasitic lice of mammals
Source: BMC Genomics. 2021 Aug 6;22:598. doi: 10.1186/s12864-021-07859-w (PMC8344215; doi:10.1186/s12864-021-07859-w)
Supplement: Supplementary file 3 — Additional file 3: PCR amplicons generated from the mitochondrial (mt) minichromosomes of the Asian grey shrew louse, Polyplax reclinata. (A) Lane 1: amplicon of S1-S2-rrnS-C minichromosome generated with the primer pair 12S364F-12S364R. Lane 2: amplicon of M-L1-rrnL-V minichromosome generated with the primer pair 16S364F-16S364R. Lane 3: 500 bp Ladder (Tiangen) (band size in bp indicated). Lane 4: amplicons of coding regions of all mt minichromosomes generated with the primer pair 364 F-364R. Lane 5: GeneRuler 100 bp DNA Ladder (Thermo Scientific) (band size in bp indicated). The lane to the right of lane 2 is irrelevant to this manuscript. The lane to the left of lane 3 is empty. (B) Lane 1: 1 kb ladder (Tiangen) (band size in bp indicated); Lanes 2–10: amplicons of individual mt minichromosomes, T-D-Y-cox2-nad6-A, R-nad4L-P-cox3, Q-nad2-N, nad1 -G-nad3-W (gene underlined has opposite transcription orientation to other genes), K-nad4, H-nad5-F, E-cob-I, cox1-L2, atp8-atp6. Details of the primers used to amplify individual mt minichromosomes are provided in Additional file 2. [file 12864_2021_7859_MOESM3_ESM.docx]

**Additional file 3.** PCR amplicons generated from the mitochondrial (mt) minichromosomes of the Asian grey shrew louse, *Polyplax reclinata*. (A) Lane 1: amplicon of *S_1_-S_2_-rrnS-C* minichromosome generated with the primer pair 12S364F-12S364R. Lane 2: amplicon of *M-L_1_-rrnL-V* minichromosome generated with the primer pair 16S364F-16S364R. Lane 3: 500 bp Ladder (Tiangen) (band size in bp indicated). Lane 4: amplicons of coding regions of all mt minichromosomes generated with the primer pair 364F-364R. Lane 5: GeneRuler 100 bp DNA Ladder (Thermo Scientific) (band size in bp indicated). The lane to the right of lane 2 is irrelevant to this manuscript. The lane to the left of lane 3 is empty. (B) Lane 1: 1 kb ladder (Tiangen) (band size in bp indicated); Lanes 2-10: amplicons of individual mt minichromosomes, *T-D-Y-cox2-nad6-A*, *R-nad4L-P-cox3*, *Q-nad2-N*, *nad1-G-nad3-W* (gene underlined has opposite transcription orientation to other genes), *K-nad4*, *H-nad5-F*, *E-cob-I*, *cox1-L_2_*, *atp8-atp6*. Details of the primers used to amplify individual mt minichromosomes are provided in Additional file 1.

**
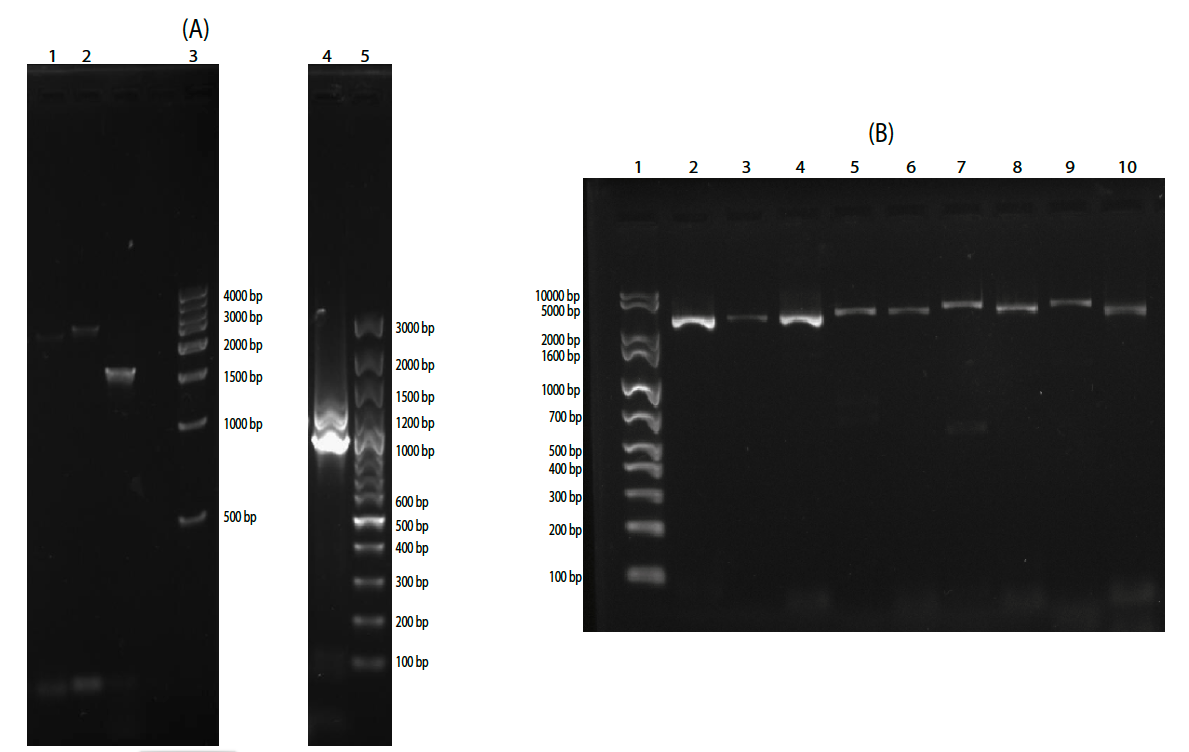
**
