# Supplementary material for: Frequent tRNA gene translocation towards the boundaries with control regions contributes to the highly dynamic mitochondrial genome organization of the parasitic lice of mammals
Source: BMC Genomics. 2021 Aug 6;22:598. doi: 10.1186/s12864-021-07859-w (PMC8344215; doi:10.1186/s12864-021-07859-w)
Supplement: Supplementary file 4 — Additional file 4: Alignment of the non-coding region (NCR) sequences of 11 mitochondrial minichromosomes of the Asian grey shrew louse, Polyplax reclinata. The primer pair, 364 F and 364R, were used to amplify the coding regions of the 11 minichromosomes (see also Additional file 3). Asterisk symbol “*” indicates conserved nucleotides; hyphen “-” indicates absent nucleotides. [file 12864_2021_7859_MOESM4_ESM.docx]

**Additional file 4.** Alignment of the non-coding region (*NCR*) sequences of 11 mitochondrial minichromosomes of the Asian grey shrew louse, *Polyplax reclinata*. The primer pair, 364F and 364R, were used to amplify the coding regions of the 11 minichromosomes (see also Additional file 2). Asterisk symbol “*” indicates conserved nucleotides; hyphen “-” indicates absent nucleotides.

**
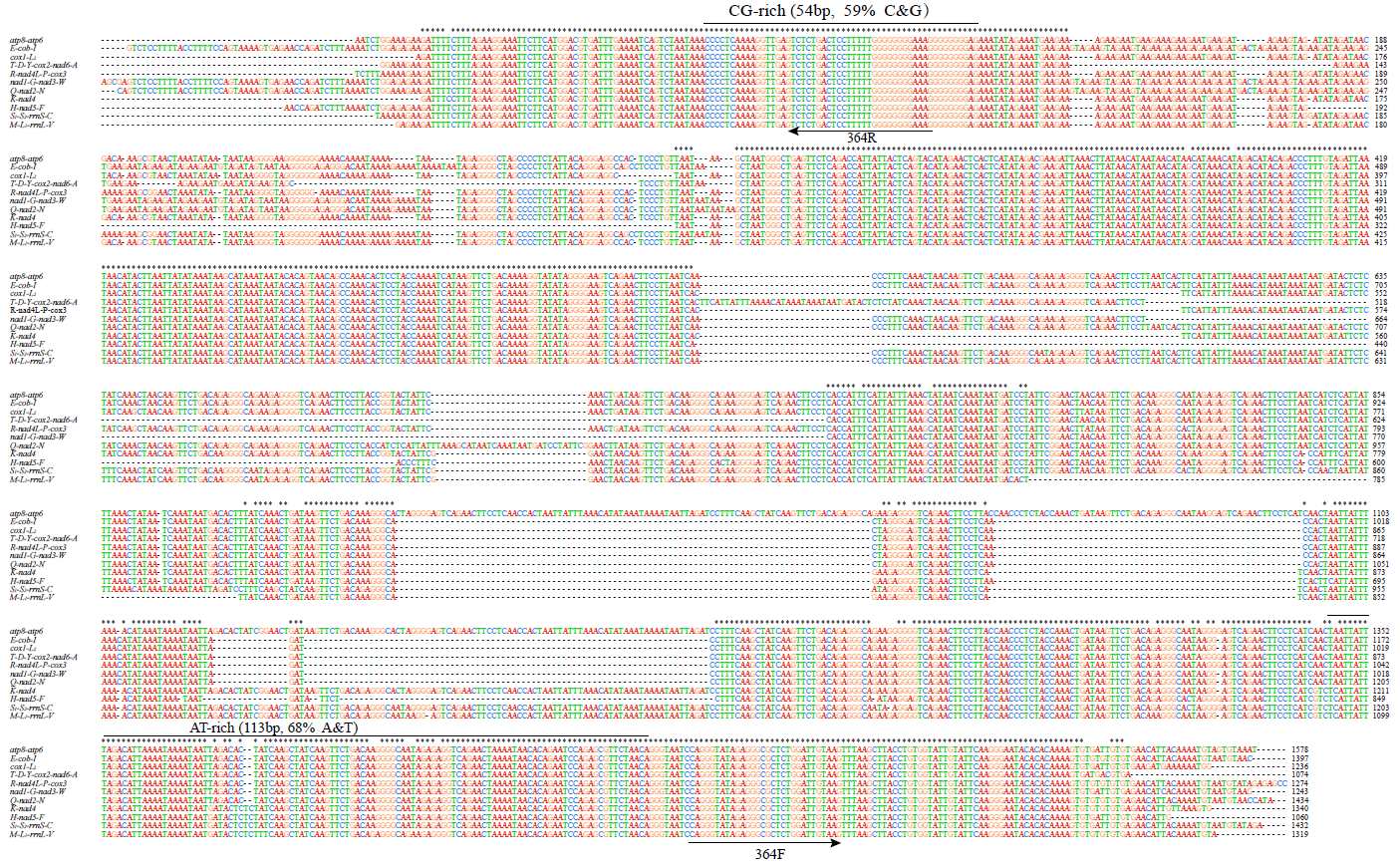
**
